# Atrophy Network Mapping of Anxiety in Cognitively Unimpaired Older Adults in Preclinical AD

**DOI:** 10.1002/alz.093939

**Published:** 2025-01-09

**Authors:** Soyoung Lee, Stephan T Palm, William J Drew, Benjamin S Zide, Nancy Donovan, Michael D Fox, Shan Siddiqi

**Affiliations:** ^1^ Brigham and Women’s Hospital, Boston, MA USA

## Abstract

**Background:**

Anxiety is prevalent among cognitively unimpaired older adults and is associated with accelerated amyloid‐ß‐related cognitive decline and incident cognitive impairment. Investigating these mechanisms is challenging due to low pathologic burden, high individual variability, and subsyndromal level of symptoms. Recently, brain networks involved in AD were successfully localized by mapping the brain connectivity of atrophy patterns associated with memory impairment and delusions. In this study, we investigated anxiety symptoms in older adults with elevated amyloid‐ß using the novel method of atrophy network mapping.

**Method:**

We utilized baseline data from ∼1800 cognitively unimpaired older adults, aged 65‐85, from the Anti‐Amyloid in Asymptomatic Alzheimer’s Disease (A4) trial, including clinical assessment of anxiety measured by the 6‐item State‐Trait Anxiety Inventory as well as structural brain MRI and amyloid‐ß (18F‐florbetapir) PET acquisitions. We generated a vertex‐wise general linear model of cortical thickness to create individualized atrophy maps for each participant with elevated amyloid‐ß deposition, using data from older adults with low amyloid‐ß deposition as a control group. Then, we used a normative human connectome (n = 1000) to estimate the functional connectivity of each participant’s unique atrophy pattern. From the network connectivity maps, we identified atrophy‐based brain networks associated with higher anxiety. To validate this brain network, we performed a spatial correlation with a pre‐established anxiosomatic network derived from focal brain stimulation sites that selectively modify anxiety and assessed significance using permutation testing. We hypothesized that atrophy patterns associated with greater anxiety would correspond to this anxiosomatic network.

**Result:**

Anxiety symptoms localized to a distinct brain network based on individuals' atrophy patterns. The identified brain network was correlated to the anxiosomatic network (r = 0.65), and the correlation was stronger when controlled for depressive symptoms (r = 0.67). The network was more similar to the anxiosomatic network than expected by chance (p<0.05).

**Conclusion:**

We identified a brain network associated with anxiety symptoms in cognitively unimpaired older adults with elevated amyloid‐ß. These findings help elucidate a neuropathophysiology of anxiety in preclinical AD and, possibly, its role in disease progression. The brain network identified in this project could yield new neurostimulation targets for anxiety in older adults across the AD continuum.

